# Conditioned medium of mesenchymal stem cells pretreated with H_2_O_2_ promotes intestinal mucosal repair in acute experimental colitis

**DOI:** 10.1038/s41598-022-24493-y

**Published:** 2022-12-01

**Authors:** Peng Liu, Xiao-ran Xie, Hao Wu, Huan Li, Jing-shu Chi, Xiao-ming Liu, Ju Luo, Yu Tang, Can-xia Xu

**Affiliations:** Department of Gastroenterology, Third Xiangya Hospital of Central South University, Changsha, 410013 Hunan China

**Keywords:** Diseases, Gastroenterology, Medical research

## Abstract

Mesenchymal stem cells (MSCs) are a new therapeutic strategy for inflammatory bowel disease (IBD), and their efficacy has been widely recognized. However, there are still some challenges in cell therapy, including stable cell passage, laboratory conditions for cell culture, high-cost burden, and poor transplantation. The conditioned medium (CM) of MSCs is considered be an excellent alternative to cell transplantation, but the paracrine group in MSC-CM is limited in variety and low in concentration, which cannot meet the therapeutic needs of injured tissues and needs to be optimized. Pretreatment with low concentration of hydrogen peroxide (H_2_O_2_) can not only protect cells from oxidative damage, but also play a role similar to growth factors and regulate the physiological function of stem cells, to obtain an improved conditioned medium. To determine the optimal protocol for pretreatment of MSCs with H_2_O_2_, and to study the efficacy and potential mechanism of MSC-CM pretreated with H_2_O_2_ on Dextran Sulfate Sodium (DSS)-induced acute experimental colitis. MSCs were exposed to different concentrations of H_2_O_2_, and the optimal H_2_O_2_ pretreatment conditions were determined by evaluating their critical cell functional properties. H_2_O_2_-pretreated MSC-CM was transplanted into experimental mouse colitis by enema at 2, 4, and 6 days in modeling, and the changes of colonic tissue structure, the levels of inflammation and oxidative stress, the molecular changes of Nrf2/Keap1/ARE axis, and the related indicators of apoptosis in colonic epithelial cells were observed in each group. In vitro, Pretreated MSCs with 25 μM H_2_O_2_ significantly enhanced cell proliferation, migration, and survival, but had no effect on apoptosis. In vivo, MSC-CM treatment decreased apoptosis and extracellular matrix deposition, and maintained the mechanical barrier and permeability of colonic epithelial cells in experimental mouse colitis. Mechanistically, H_2_O_2_-pretreated MSC-CM against reactive oxygen species (ROS) production and MDA generation, accompanied by increases in components of the enzymatic antioxidant system includes SOD, CAT, GSH-PX, and T-AOC, which is through the up-regulation of the Nrf2, HO-1, and NQO-1 antioxidant genes. Our data confirmed that 25 μM H_2_O_2_ pretreated MSC-CM treatment could effectively improve intestinal mucosal repair in experimental colitis, which may be achieved by activating Nrf2/Keap1/ARE pathway.

## Introduction

Inflammatory bowel diseases (IBD) are chronic inflammatory conditions that affect the gastrointestinal tract, mainly encompassing Crohn’s disease (CD) and ulcerative colitis (UC)^1^. So far, the etiology of IBD has not been clearly identified, and it is generally believed to be caused by a disordered mucosal immune response to environmental factors in genetically susceptible hosts^2^. IBD predominantly affects young individuals, with a prevalence in western countries up to 0.5% of the general population, with growing incidence^5^, and these patients have a higher risk of colon cancer due to persistent chronic inflammation, as well as a higher mortality rate^6^. Despite some drug advances, nearly 30% of patients do not respond to current treatments, and 50% suffer allergic reactions or become refractory over time^5^. Therefore, it is necessary to explore novel strategies to strengthen the therapeutic capacity of IBD.

Mesenchymal stem cells (MSCs) are a type of adult stem cell, that can be isolated from a variety of tissues. Over the past decade, MSCs have attracted much attention in the field of regenerative medicine. MSCs can migrate to the sites of inflammation and hold potent of immunomodulatory and anti-inflammatory effects through cell and cell interactions between MSCs and lymphocytes or production of soluble factors^8^. A meta-analysis included eight animal (n = 132) and seven human (n = 216) trials published in 2019 suggested that MSCs therapy shows safety and efficacy for the treatment of IBD^12^. Given the excellent performance of MSCs in these scientific studies and clinical trials, MSCs are being explored as a possible therapeutic alternative. Although MSCs are currently the most widely used stem cells, several challenges remain in cell therapy, including achieving stable cell passaging, creating laboratory conditions for stem cell culture, identifying the optimal mode of cell delivery, and a high-cost burden. Some reports suggest that the therapeutic effects of MSCs in injured tissue are mainly mediated by paracrine activity, which shows that conditioned medium (CM) generated from MSCs also has some therapeutic potential. Chen et al. suggested that the CM of MSCs (MSC-CM) could reduce irradiation-induced TGF-β1 production through inhibiting the NF-κB signaling pathway, thereby alleviating irradiation-induced damage in cardiac fibroblast cells^10^. In a rat model of full-thickness skin defect wound, a Hypoxia-pretreated rat MSC-CM can accelerate wound healing by regulating inflammatory cell infiltration, promoting re-epithelialization and collagen deposition^11^. Similarly, Xu et al. suggested that MSC-CM can improve islet viability and function by increasing autophagy through the PI3K/Akt/mTOR pathway under hypoxic conditions^12^. Growing evidence indicates that MSCCM-based therapy holds excellent therapeutic value.

Our study pretreated MSCs with H_2_O_2_, because many studies demonstrated that low concentration H_2_O_2_ pretreatment could protect cells from oxidative damage and play a role similar to growth factors in regulating physiological stem cellular processes. For example, Guo et al. observed in the wound model that the wound micro-vessel density increased and wound closed rapidly after the transfusion of H_2_O_2_-pretreated MSCs^13^. The existence of oxidative stress is of great significance to the occurrence and development of IBD. Oxidative stress is a state of imbalance between oxidation and anti-oxidation in the body. When the neutrophils and macrophages in the intestinal mucosa reach the damaged part of the intestine, under the action of the reduced coenzyme and oxidase of the cell membrane, a large number of superoxide anion (O_2_^−^), hydroxyl radicals (OH) and lipid peroxides are produced through cell respiration, leading to the occurrence of oxidative stress. There are two types of antioxidant systems in the body: a non-enzymatic antioxidant system and an enzymatic antioxidant system that includes antioxidant enzymes catalase (CAT), superoxide dismutase (SOD), glutathione peroxidase (GSH-PX), and antioxidation capacity (AOC), which protect cells from damage caused by oxidative stress. Nuclear factor erythroid-derived 2-like 2- (Nrf2-) Kelch-like ECH-associated protein 1- (keap1-) antioxidant response element (ARE) signaling pathway-it is one of the most important pathways of antioxidant stress, which plays a vital role in the balance between oxidative system and antioxidant system in the body^14^.

Our study determined the optimal concentration of H_2_O_2_ for the pretreatment of MSCs. Combined with Nrf2/Keap1/ARE pathway, the therapeutic potential and molecular mechanism of H_2_O_2_-pretreated MSC-CM were evaluated in a mouse experimental colitis model.

## Materials and methods

### Cell culture and CM collection

MSCs were derived from the bone marrow of C57BL/6 mice and purchased from Cas9X™ Biosciences, Inc. (Suzhou, China). MSCs were cultured in a special medium supplemented with 10% MSC qualified fetal bovine serum (FBS), 100 U/mL penicillin/streptomycin, and 1% glutamine (all purchased from Cas9X™ Biosciences, Inc.) at 37 °C. The medium was replenished every 24–48 h. When cells were 80–90% confluent, they were isolated with TrypLE reagent to obtain a cell suspension. MSCs were collected and centrifuged at 800*g* for 3–5 min at room temperature, then re-suspended in a fresh culture medium and counted using a light microscope.CM was aspirated following a minimum of 24 h incubation with MSCs and used for in vivo experiments. Similarly, MSCs and H_2_O_2_ were co-cultured for at least 24 h before the obtained MSC-CM could be used in animal experiments.

### Flow cytometry analysis of MSCs apoptosis

Cell apoptosis was detected with Annexin V-Fluorescein Isothiocyanate (FITC)/Propidium Iodide (PI) Apoptosis Detection Kit (Beyotime Biotechnology, Jiangsu, China) as described by the manufacturer’s instructions^15^. MSCs were seeded in 6-well plates at a density of 1 × 10^5^ cells/well, and treated with various concentrations of H_2_O_2_ (25, 50, 100, 200, and 400 μM) for 24 h. After that, cells were collected and washed with ice-cold PBS twice, gently re-suspended in the binding buffer and incubated with annexin V-FITC and PI for 15 min in the dark. Cells were analyzed by flow cytometry. The total apoptosis rate of cells was calculated as the sum of the rates of cells observed in the lower-right quadrant and the upper-right quadrant.

### Detection of cell activity by CCK-8

After cells were treated with H_2_O_2_, these cells were washed three times with PBS, and 100 μL of a complete medium and 10 μL of CCK-8 solution (Beyotime Biotechnology, Jiangsu, China) were added to each well. After incubation for 2 h, the enzyme-linked immunosorbent assay was performed at 450 nm. Then, the absorbance value (OD) was measured.

### Cell ATP assay

Because mitochondrial activity can better reflect cell vitality, cell viability was assessed using the ATP assay. A luciferase-based kit (Beyotime Biotechnology, Jiangsu, China) was used to measure the ATP level according to the manufacturer’s instructions. Cells were treated with various concentrations of H_2_O_2_ for 24 h at 37 °C in a cell culture incubator. Next, the supernatant was quickly mixed with the working solution at equal volumes and then transferred into a standard opaque well 96-well plate before light recording a microplate reader using the chemiluminescence method.

### Animals and induction of DSS-induced UC

Male and female C57BL/6 mice (6–8 weeks old) were purchased from the Experimental Animals Department of Central South University (Changsha, China). Mice were divided into different groups (n = 6 animals per group per time point). Experimental colitis was induced in C57BL/6 mice by continuous administration of 2% DSS (MW: 36,000–50,000, MP Biomedicals, Santa Ana, CA) in drinking water for 7 days^16^. In the control group, mice were given normal drinking water. The mice in the MSC-CM group underwent enema administration of 200 µL CM, those in the MSC-CM + H_2_O_2_ group were treated with 200 µL CM of MSCs pretreated with H_2_O_2_ for 24 h, and those in the MSC-CM + H_2_O_2_ + ML385 group were intraperitoneally injected with ML385 (30 mg/kg daily, a novel and specific inhibitor of) in addition to the MSC-CM + H_2_O_2_ therapy. The mice in each group were additionally anesthetized with isoflurane on the 2nd, 4th, and 6th day for treatment. After enema treatment, the mice were held at an inverted angle to ensure that the therapeutics did not leak from the rectum, and the procedure was maintained for at least 30 min. During this period, body weight, stool consistency, and rectal bleeding symptoms were monitored and recorded daily, and the disease activity index (DAI) was developed based on these data. The mice were sacrificed on day 8 to measure colon length and spleen weight. A portion of the distal colon tissue was harvested and stored for further histological testing.

### Pathological assessment

The paraffin-embedded sections were stained using protocols for hematoxylin and eosin (H&E) and Masson’s trichrome staining. The histological severity of colitis was observed under a microscope, and the histological score of H&E-stained tissue samples was based on the degree of edema, ulcers, crypt loss, and immune cell infiltration^17^. Masson’s trichrome staining was used to assess collagen deposition in the colonic stroma, and ImageJ software was used to analyze the collagen area ratio.

### ROS levels and antioxidant enzymes

Cells were centrifuged and supernatant extracted after cell lysate was used for detection. Colon tissues were poured into a glass homogenizer, and an appropriate amount of normal saline was added for grinding to prepare a homogenate. The protein concentration was determined by the BCA assay. ROS-related indexes includes superoxide anion (O_2_^−^), hydroxy radical (OH), and malondialdehyde (MDA), and antioxidant enzymes related indexes includes antioxidant enzymes catalase (CAT), total superoxide dismutase (T-SOD), glutathione peroxidase (GSH-PX), and total antioxidation capacity (T-AOC) activity in colonic tissues were tested using a relevant assay kit (Nanjing Jiancheng Bioengineering Institute, Nanjing, China).

### Cytokine measurements

To identify the concentrations of IL-1β, TNF-α (Boster Biological Technology Co., Ltd., California, USA), IL-6, and IL-10 (Elabscience Biotechnology Co. Ltd, Wuhan, China), an enzyme-linked immunosorbent assay (ELISA) for each cytokine were performed according to the manufacturer’s instructions^18^.

### Western blot analysis

Apoptosis- and Nrf2/Keap1/ARE signaling-related proteins were detected using western blotting. Total protein was separated using 10% SDS-PAGE and transferred to a polyvinylidene difluoride (PVDF) membrane. The membrane was blocked with TBS-T containing 5% low-fat dried milk for 1 h at room temperature. The membrane was then incubated with primary antibodies (specific for Nrf2, Keap1, HO-1, NQO-1, Bax, Bcl-2, Caspase 3, Cleaved Caspase3, ZO-1and GAPDH) overnight at 4 °C. Horseradish peroxidase-conjugated anti-rabbit secondary antibodies (Wuhan Boster Biological Technology, Wuhan, China) at a 1:5000 dilution were incubated with the membrane at room temperature for 1 h. The blot was imaged with the chemiluminescent imager, and then ImageJ was used to analyze the gray value of the images^19^.

### Immunohistochemical staining

All paraffin sections were pretreated and incubated with an anti-CD68 rabbit polyclonal antibody (1:200) and anti-MPO rabbit polyclonal antibody (1:100) at 4 °C under humid conditions overnight. Each slide was incubated with secondary biotinylated anti-mouse IgG, followed by activin/biotin ABC reagent reaction.

### Immunofluorescence (IF) assays

For immunofluorescence staining, sections were incubated with mouse monoclonal antibodies against ZO-1, NF-κB p65, and FOXP3 (1:200) and subsequently with FITC-conjugated secondary antibodies. 4′,6-Diamidino-2-phenylindole (DAPI) was utilized as a nuclear stain, and images were captured under a Leica DMIRE2 confocal laser scanning microscope.

### Statistical analysis

Statistical analyses were performed using SPSS statistical software (version 23.0). One-way analysis of variance followed by Bonferroni’s post hoc test was performed for multiple-group comparisons. If the P value was less than 0.05 (*), 0.01 (**), or 0.001 (***), the difference was considered significant.

### Compliance with ethical standards

All the protocols were approved by the Ethics Committees at the Department of Experimental Animals of Central South University. All experimental procedures strictly adhered to the principles established by the Department of Experimental Animals of Central South University. The study was carried out in compliance with the ARRIVE guidelines.

## Results

### Low concentration H_2_O_2_ preconditioning significantly enhanced cell viability and proliferation without affecting cell apoptosis

**Figure 1:**
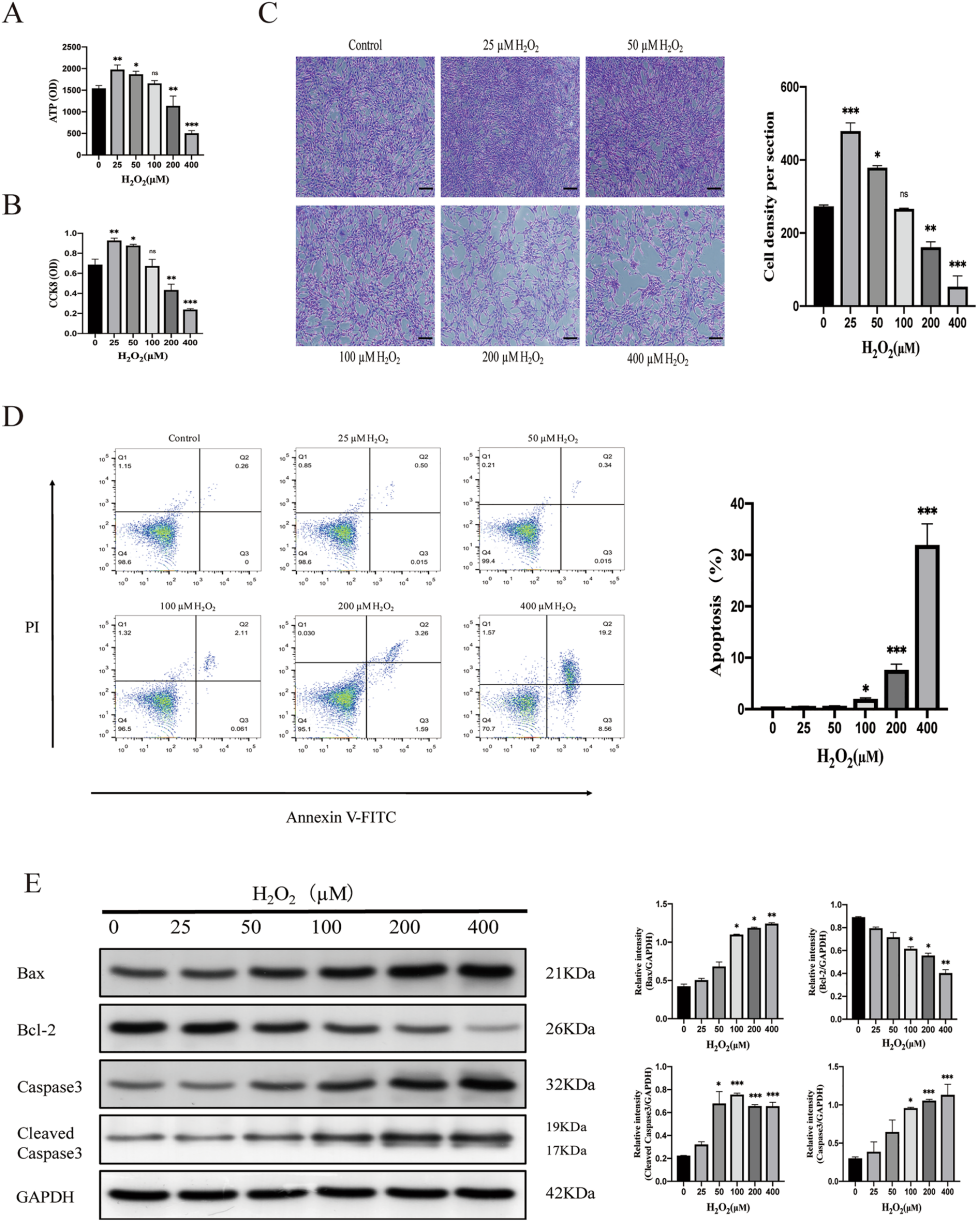
Optimization of H_2_O_2_ concentrations for preconditioning MSCs. (**A**) Cell viability was analyzed by ATP assay. (**B**) The effect of H_2_O_2_ on MSCs proliferation was determined by the CCK-8 assay. (**C**) Crystal violet staining assessed the cell density are shown, quantitative analysis of cell density. Magnification: × 40. Scale bar, 200 μm. (**D**) The apoptosis of MSCs treated with different concentrations of H_2_O_2_ for 24 h was analyzed by flow cytometry. The bottom right quadrant represents annexin V-FITC-stained cells (early-phase apoptotic cells), and the top right quadrant represents PI- and annexin V-FITC-dual-stained cells (late-phase apoptotic/necrotic cells). Apoptotic cells are represented as the percentage. (**E**) Western blot analysis of Bcl-2, Bax, Caspase 3, and Cleaved Caspase3 in MSCs exposed to 25–400 μM H_2_O_2_ for 24 h, and semi-quantitative analysis of the related proteins. Data are expressed as the mean ± SD (n = 3). *P < 0.05, **P < 0.01, and ***P < 0.001; *ns *no significance.

To determine the optimal concentration of H_2_O_2_ in pretreated MSCs, we selected five different concentrations in the range of 25–400 μM according to previous reports. We studied their effects on cellular properties of viability, proliferation, and apoptosis. By using the ATP assay, CCK-8 assay, and crystal violet staining, we found that 25 μM and 50 μM H_2_O_2_ significantly increased the viability of MSCs (Fig. 1A) and promoted the proliferation of MSCs (Fig. 1B,C), but the concentration of H_2_O_2_ at 200 and 400 μM has an apparent inhibitory effect. Further, Flow Cytometric (FCM) analysis showed that exposure to 25–50 μM H_2_O_2_ did not induce apoptosis of MSCs (Fig. 1D). However, compared with the control group, exposure to 100–400 μM H_2_O_2_ induced a significant increase in apoptosis rates of MSCs in a dose-dependent manner. The results showed that H_2_O_2_ concentration above 100 μM could cause oxidative damage and apoptosis of cells.

Oxidative stress induces apoptosis through the intrinsic mitochondrial pathway^20^. The ratio of anti-apoptotic Bcl-2 and pro-apoptotic Bax (Bcl-2/Bax) is the key to regulating the mitochondrial apoptotic pathway [30]. After 25 μM H_2_O_2_ treatment, the Bcl-2/Bax expression ratio was not affected compared with the control group (Fig. 1E). With the concentration above 50, the ratio of Bcl-2/Bax decreased, and the expression of Bax, Caspase-3 and Cleaved Caspase3 increased gradually. Therefore, 25 μM H_2_O_2_ may be the best condition for MSCs pretreatment in vitro from the expression ratio of anti-apoptotic and pro-apoptotic protein.

### Pretreatment of MSCs with 25 μM H_2_O_2_ could attenuate oxidative stress-induced cell death by enhancing Nrf2/Keap1/ARE signaling

The production of ROS increases or the ability of the body to clear ROS decreases, which can result increasing of lipid peroxidation level of body tissue, DNA oxidative damage, and abnormal protein expression. Nrf2/Keap1/ARE signaling pathway is one of the most important cellular defense mechanisms against oxidative stress, and Nrf2 is an essential protective transcription factor, which mediates energy metabolism and regulates cell cycle and autophagy by regulating the expression of genomes related to detoxification, antioxidant defense and cell protection^21^.

**Figure 2:**
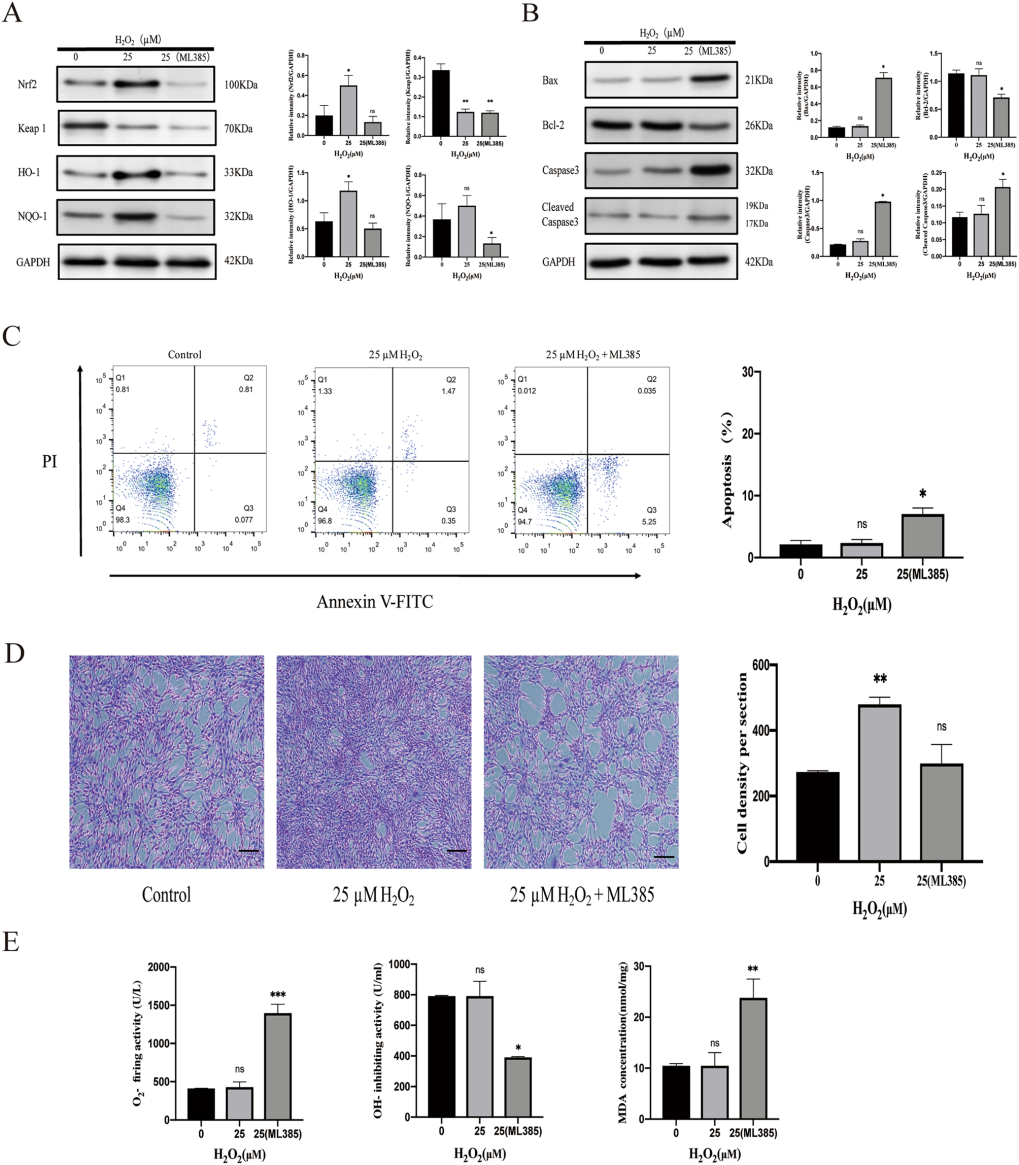
25 μM H_2_O_2_ pretreatment of MSCs can reduce apoptosis caused by ROS generation by activating the Nrf2/Keap1/ARE signaling pathway. (**A**) Immunoblotting assay determined the abundance of Nrf2, Keap1, HO-1, and NQO-1 proteins, and semi-quantitative analysis of the related proteins. (**B**) Immunoblotting assay determined the abundance of Bax, Bcl-2, Caspase 3, and Cleaved Caspase3 proteins, and semi-quantitative analysis of the related proteins. (**C**) Representative images of flow cytometric analysis by annexin V-FITC/PI dual staining. Apoptotic cells are represented as the percentage. (**D**) Crystal violet staining assessed the cell density are shown, quantitative analysis of cell density. Magnification: × 40. Scale bar, 200 μm. (**E**) The quantitative evaluation of oxidative stress in MSCs including O_2_^−^, OH^−^, and MDA. The data are expressed as the mean ± SD (n = 3). *P < 0.05, **P < 0.01, and ***P < 0.001; *ns* no significance.

We found that pretreatment of MSCs with 25 μM H_2_O_2_ could activate the intracellular Nrf2/Keap1/ARE signaling pathway, and the expression levels of Nrf2 and its downstream proteins HO-1 and NQO-1 were significantly increased, which was reversed after the use of Nrf2 inhibitor ML385 (Fig. 2A). Pretreatment with low dose of H_2_O_2_ did not affect the expression of apoptosis-related proteins, but after the use of Nrf2 inhibitor ML385, Bax, caspase-3 and Cleaved Caspase3 were significantly increased and Bcl-2 was decreased (Fig. 2B), indicating that the cells were under apoptosis, which was consistent with the results of flow cytometry (Fig. 2C). In addition, crystal violet staining showed that the proliferation of MSCs pretreated with 25 μM H_2_O_2_ was also inhibited after the use of Nrf2 inhibitor ML385 (Fig. 2D). We also assessed ROS production and MDA (an indicator reflecting the degree of lipid peroxidation) generation, suggesting that the use of Nrf2 blockers contributes to intracellular oxidative stress (Fig. 2E).

In conclusion, Nrf2/Keap1/ARE signaling pathway plays a vital role in mediating cell apoptosis and reducing oxidative stress.

### Therapeutic effects of MSC-CM enema in experimental colitis mice

**Figure 3:**
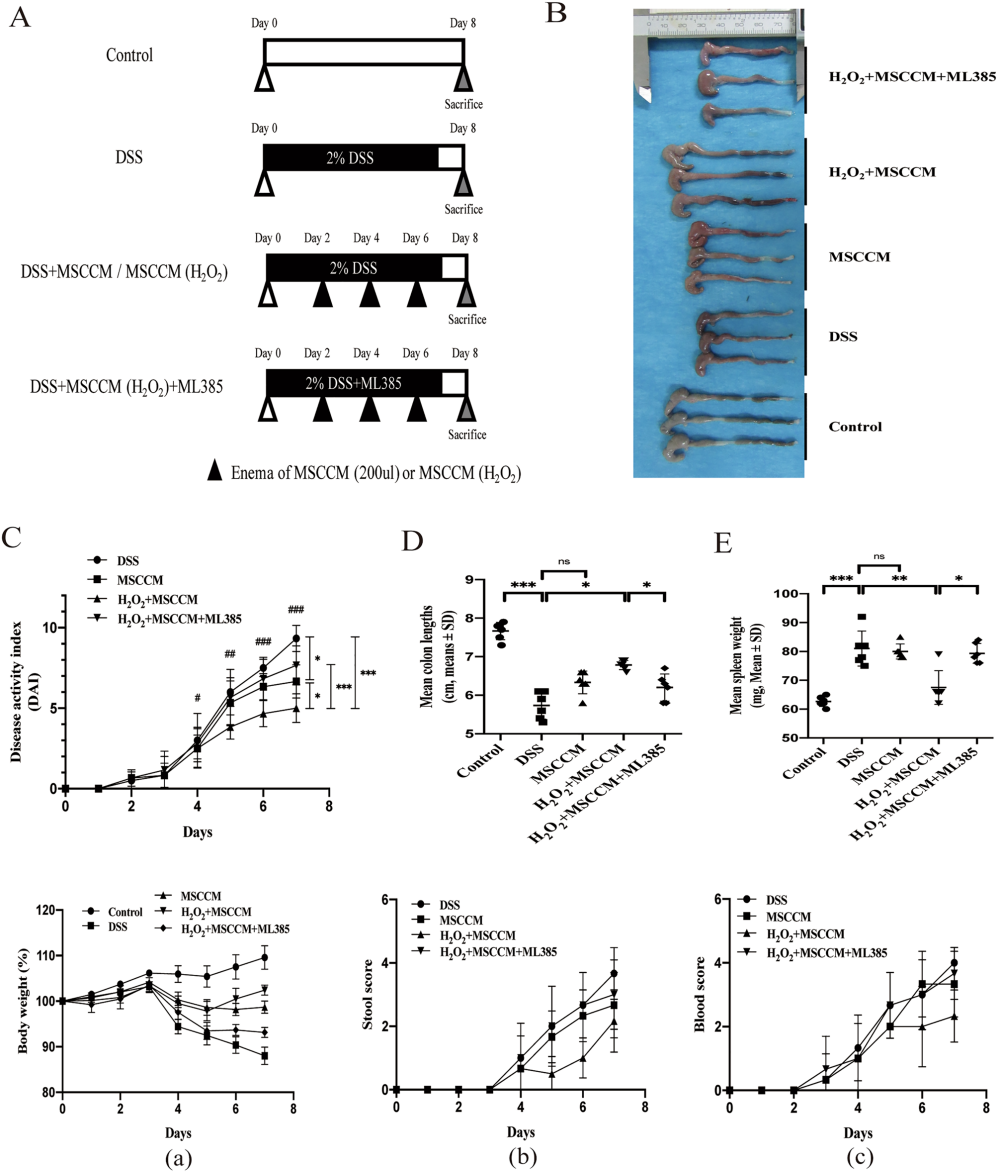
Therapeutic effects of H_2_O_2_ pretreated MSC-CM in DSS-induced colitis mice. (**A**) Experimental protocol for DSS-colitis model. Mice received drinking water with 2% DSS for 7 days, and treatment groups were given an enema administration of vehicle control, 25 μM H_2_O_2_ pretreated MSC-CM (200 µl), MSC-CM (200 µl), or ML385 (30 mg/kg). (**B**) Macroscopic images of representative colons at sacrifice. (**C**) Disease activity score is based on the loss of body weight (a), stool condition (b), and fecal occult blood (c) at sacrifice. (**D**) Disease-related shortening of the colon and quantified in a bar graph. (**E**) Disease-related lightening of the spleen and quantified in a bar graph. The data are expressed as the mean ± SD. n = 6 animals per group per time point. *P < 0.05, **P < 0.01, and ***P < 0.001, significantly different between groups; ns = no significance. ^#^P < 0.1, ^##^P < 0.01, ^###^P < 0.001, significantly different within groups.

The symptoms of DSS-induced colitis were similar to human UC, such as a shortened colon, weight loss, and bloody diarrhea. MSC-CM and 25 μM H_2_O_2_-preconditioned MSC-CM were administered via the anus on day 2, day 4 and day 6 according to the experimental protocol shown in Fig. 3A. The comparison of colonic appearance in each group was shown in Fig. 3B, and the DAI scores of treated mice was significantly reduced compared with those of DSS-induced colitis mice (Fig. 3C). It should be noted that although treatment with MSC-CM could effectively improve DAI scores, it was less effective than 25 μM H_2_O_2_-preconditioned MSC-CM therapy and could not prevent the significant reductions in colon length (Fig. 3D) and spleen weight (Fig. 3E). Further study showed that the protective effects of 25 μM H_2_O_2_-preconditioned MSC-CM on DSS-induced colitis were counteracted by pretreatment with an Nrf2 inhibitor (ML385).

### MSC-CM treatments alleviate histopathological symptoms in DSS-induced experimental colitis

**Figure 4:**
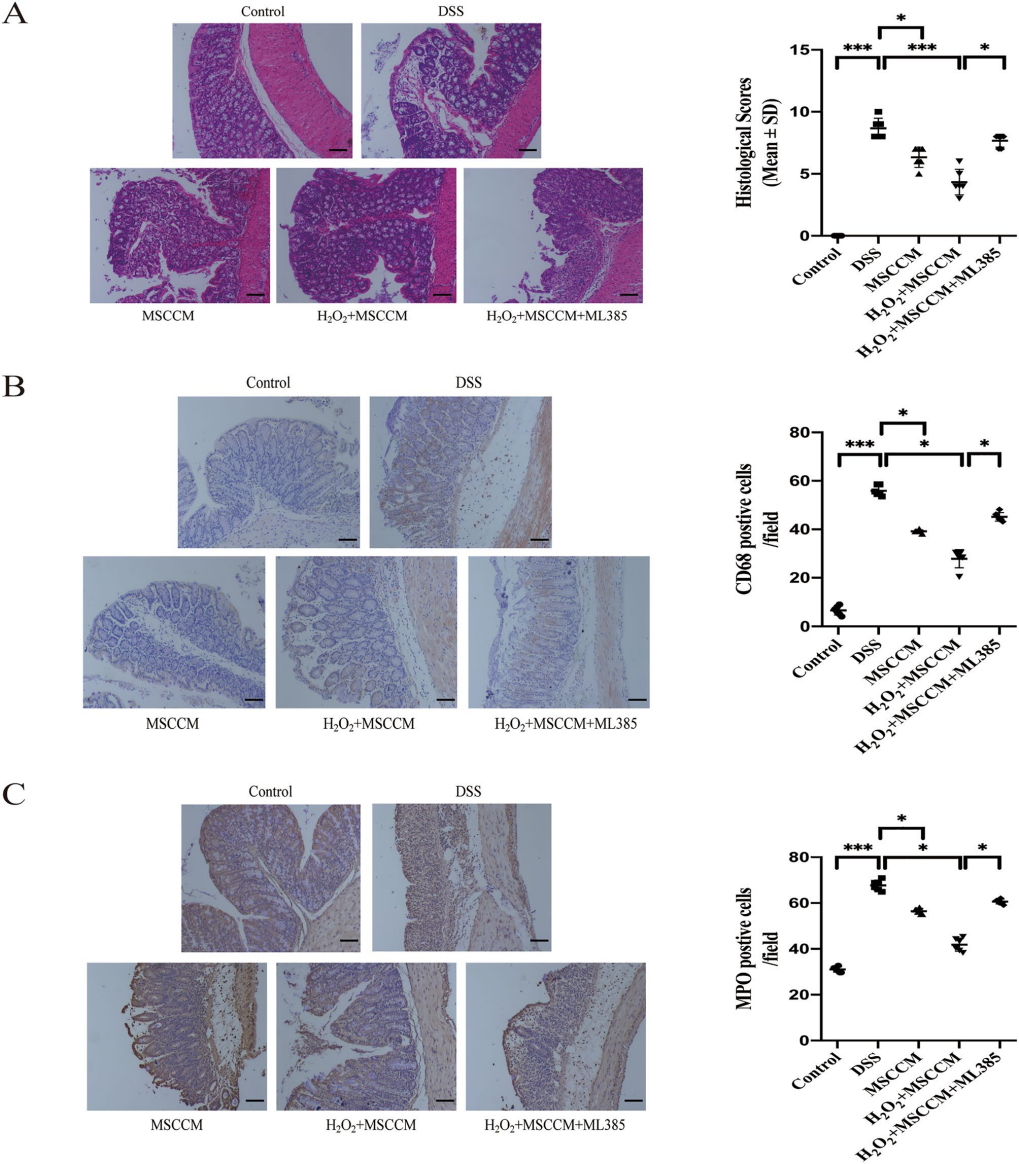
H_2_O_2_ pretreated MSC-CM decreases the inflammatory cell in colon tissue. (**A**) Distal colons were removed and sectioned followed by H&E staining. Representative sections are displayed. Inflammation scores were evaluated in bar graph. (**B**) IHC analysis for the macrophage specific marker CD68 was performed, and subsequently positive cells were evaluated. Magnification: × 100. Scale bar, 100 μm. (**C**) IHC analysis for the neutrophil specific marker MPO was performed, and subsequently positive cells were evaluated. The insets are magnified images of the indicated rectangles. Magnification: × 100. Scale bar, 100 μm. Data are expressed as the mean ± SD. n = 6 animals per group per time point. *P < 0.05, **P < 0.01, and ***P < 0.001; *ns* no significance.

Whether MSC-CM was pretreated with H_2_O_2_- or not, H&E staining of colon sections of sacrificed mice showed that they reduced the infiltration of inflammatory cells and the inflammation scores in the distal colon compared with those of DSS-induced colitis mice (Fig. 4A). Next, we tested the activities of MPO (a marker of neutrophils) and CD68 (a marker of macrophages), MSC-CM can reduce neutrophil and macrophage infiltration in both MSC-CM treatment groups (Fig. 4B,C). Likewise, Pretreatment of MSCs with 25 μM H_2_O_2_ has a better anti-inflammatory effect, but the curative effect will be offset by adding of ML385.

### MSC-CM treatments reduced the infiltration of inflammatory in DSS-induced experimental colitis

**Figure 5:**
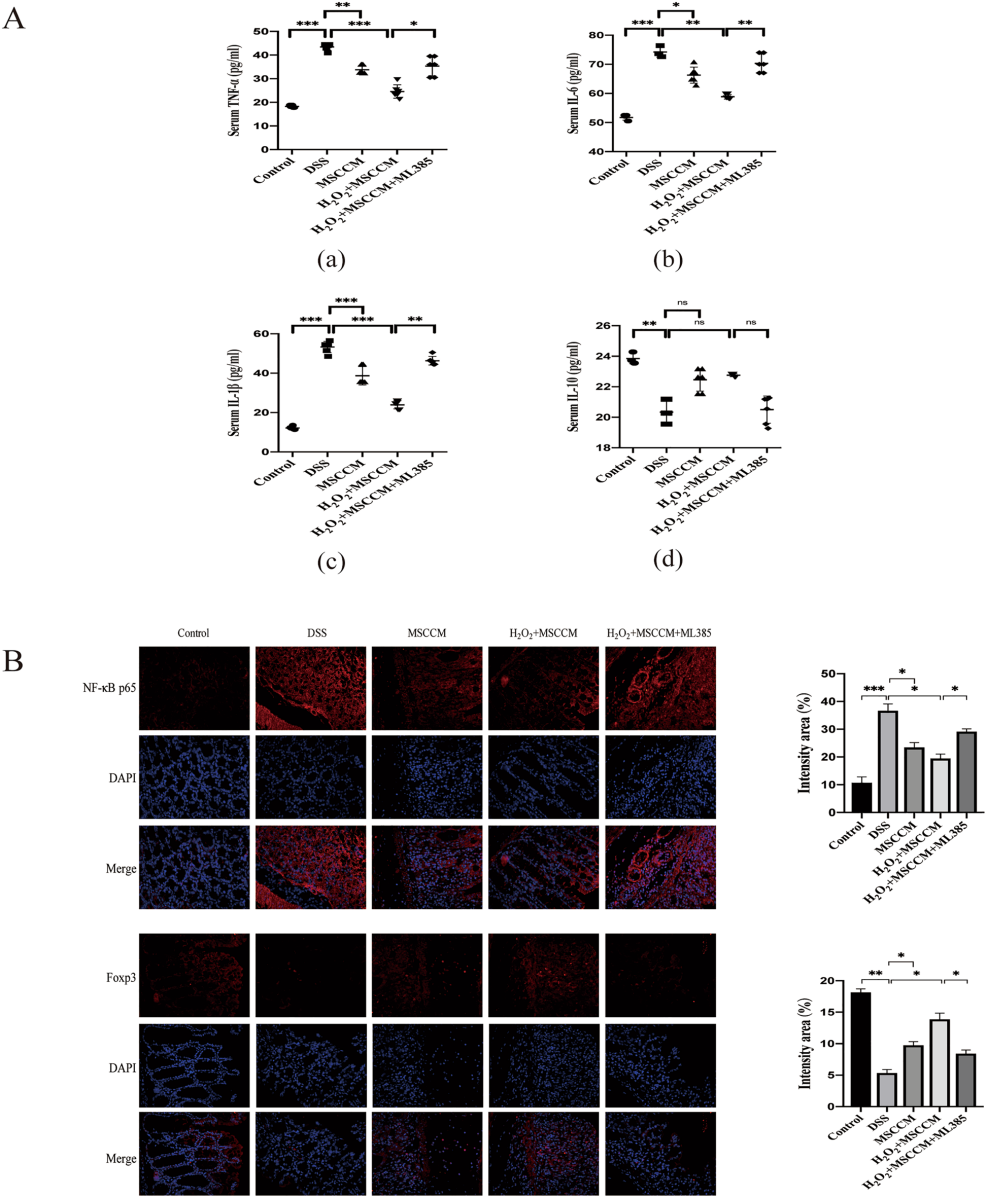
H_2_O_2_ pretreated MSC-CM regulates the inflammatory cytokines in colon tissue. (**A**) The levels of inflammatory cytokines including TNF-α (a), IL-6 (b), IL-1β (c), and IL10 (d) in colon tissue homogenate were detected by ELISA. Data are expressed as the mean ± SD. n = 6 animals per group per time point. *P < 0.05, **P < 0.01, and ***P < 0.001; *ns* no significance. (**B**) Sections of colonic tissues were immunostained for revealing NF-κB p65 proteins (red) and Foxp3 proteins (red) as indicated. The slides were counterstained with DAPI (blue), and the images were captured using an inverted fluorescence microscope. Magnification: × 400.

We measured the levels of inflammatory cytokines such as IL-1β, TNF-α, IL-6, and IL-10 (Fig. 5A), which are linked to colitis. The DSS group showed increased levels of the pro-inflammatory cytokines IL-1β, TNF-α, and IL-6, whereas the levels of these pro-inflammatory cytokines were decreased after MSC-CM treatments. The concentration of IL-10, a recognized suppressor of inflammation and immunity, was reduced in the colitis group, but there was no difference among the treatment groups. Considering that IL-6 can inhibit TGF-β-induced regulatory T cell (Treg) differentiation and plays a significant role in maintaining the balance between T helper 17 (Th17) cells and Tregs, we performed immunofluorescence staining for Foxp3 + Tregs (Fig. 5B), the results clearly showed that MSC-CM could increase the level of Foxp3 + Tregs. We also evaluated the expression of nuclear transcription factor-κB (NF-κB), a classical key nuclear transcription factor, which is involved in the regulation of the above-mentioned inflammatory factors. Compared with the DSS group, the treatment of MSC-CM can significantly reduce NF-κB expression. It is worth noting that 25 μM H_2_O_2_-preconditioned MSC-CM therapy is more effective than the MSC-CM group, and the effect also disappeared with the addition of ML385.

### MSC-CM ameliorates intestinal mucosal barrier injury in DSS-induced experimental colitis

**Figure 6:**
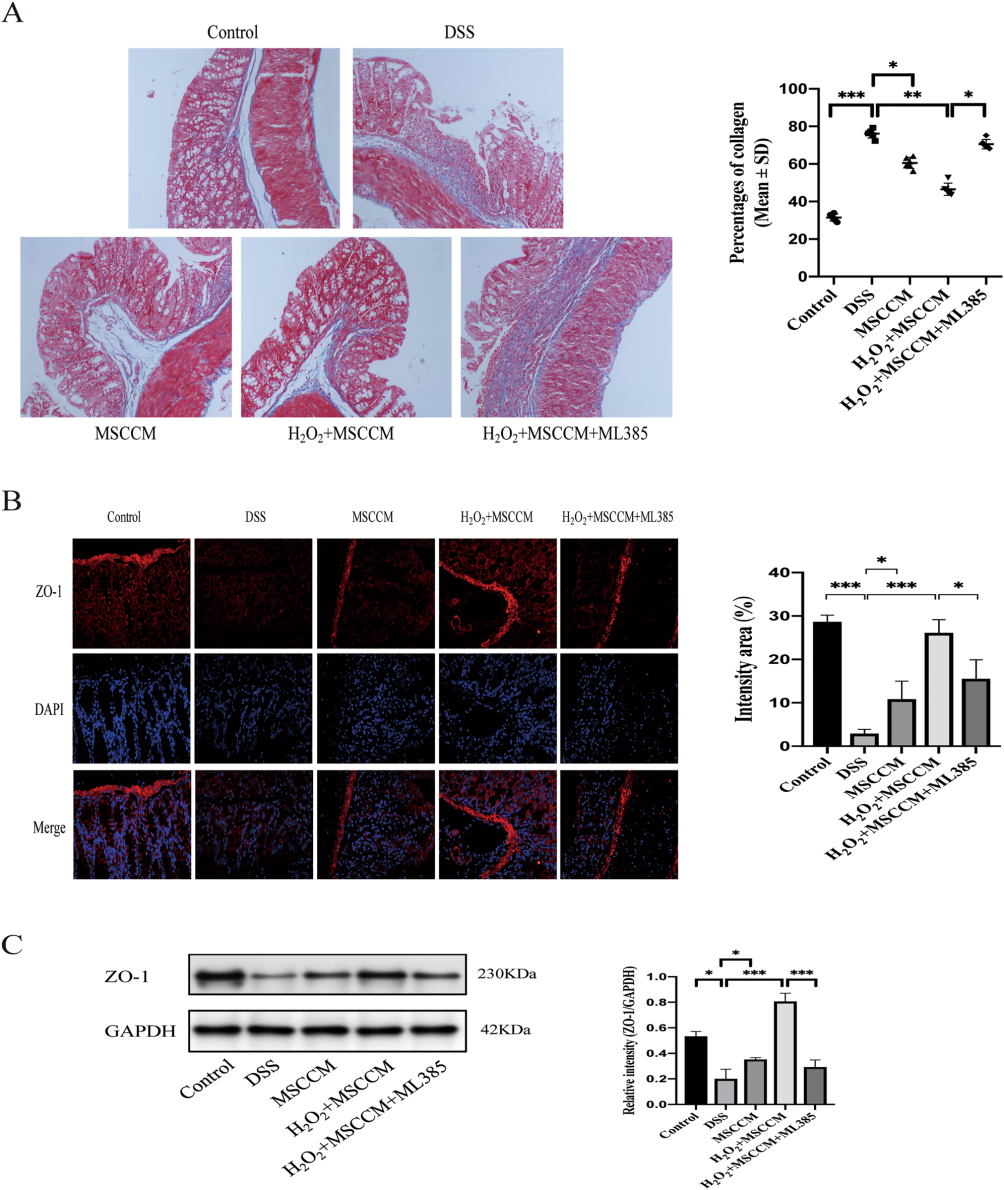
H_2_O_2_ pretreated MSC-CM reduces intestinal mucosal barrier injury in colon tissue. (**A**) Masson staining assessed the collagen deposition in the colonic interstitium, the collagen area ratio was evaluated in bar graph. The insets are magnified images of the indicated rectangles. Magnification: × 100. Scale bar, 100 μm. Values are the means ± SD. n = 6 animals per group per time point. *P < 0.05, **P < 0.01, and ***P < 0.001; *ns* no significance. (**B**) Sections of colonic tissues were immunostained for revealing ZO-1 proteins (red) as indicated. The slides were counterstained with DAPI (blue), and the images were captured using an inverted fluorescence microscope. Magnification: × 400. (**C**) Immunoblotting assay determined the abundance of ZO-1 proteins. Semiquantitative analysis of proteins of interest by densitometry assay. The data are expressed as the mean ± SD. *P < 0.05, **P < 0.01, and ***P < 0.001; *ns* no significance.

Masson’s trichrome staining showed that only a small amount of collagen was deposited in the mucosa and submucosa in the control group. In contrast, a large amount of collagen was deposited in the mucosa, submucosa, and muscle layer of the intestinal wall in DSS-induced colitis. The collagen deposition observed after administration of MSC-CM enema was less than that observed in the DSS group. Interestingly, the presence of an inhibitor of Nrf2 (ML385) eliminated the therapeutic effect of H_2_O_2_-preconditioned MSC-CM. The collagen area ratio was analyzed by ImageJ software, the collagen areas of the control and treated groups were significantly smaller than that of the DSS group, and the differences were statistically significant (Fig. 6A).

We further using immunofluorescence and western blot evaluated the ZO-1 protein (Fig. 6B,C), a crucial component of tight junctions. Downregulation of ZO-1 expression or activity affects the formation of tight junctions between cells, hinders the intestinal mucosa from playing a vital barrier function in host defense, and increases the risk of enterogenous infection caused by harmful bacteria and toxins penetrating the intestinal tract. Compared to the control group, we observed that the ZO-1 protein expression in intestinal epithelial cells was significantly reduced in the DSS group. After MSC-CM enema treatment, the expression of ZO-1 was notably improved, and the 25 μM H_2_O_2_ -preconditioned MSC-CM therapy was more effective. Similarly, with the addition of ML385, the expression of ZO-1 was significantly decreased.

### 25 μM H_2_O_2_-preconditioned MSC-CM decrease cell apoptosis and oxidative stress via activation of the Nrf2 pathway in DSS-induced experimental colitis

**Figure 7:**
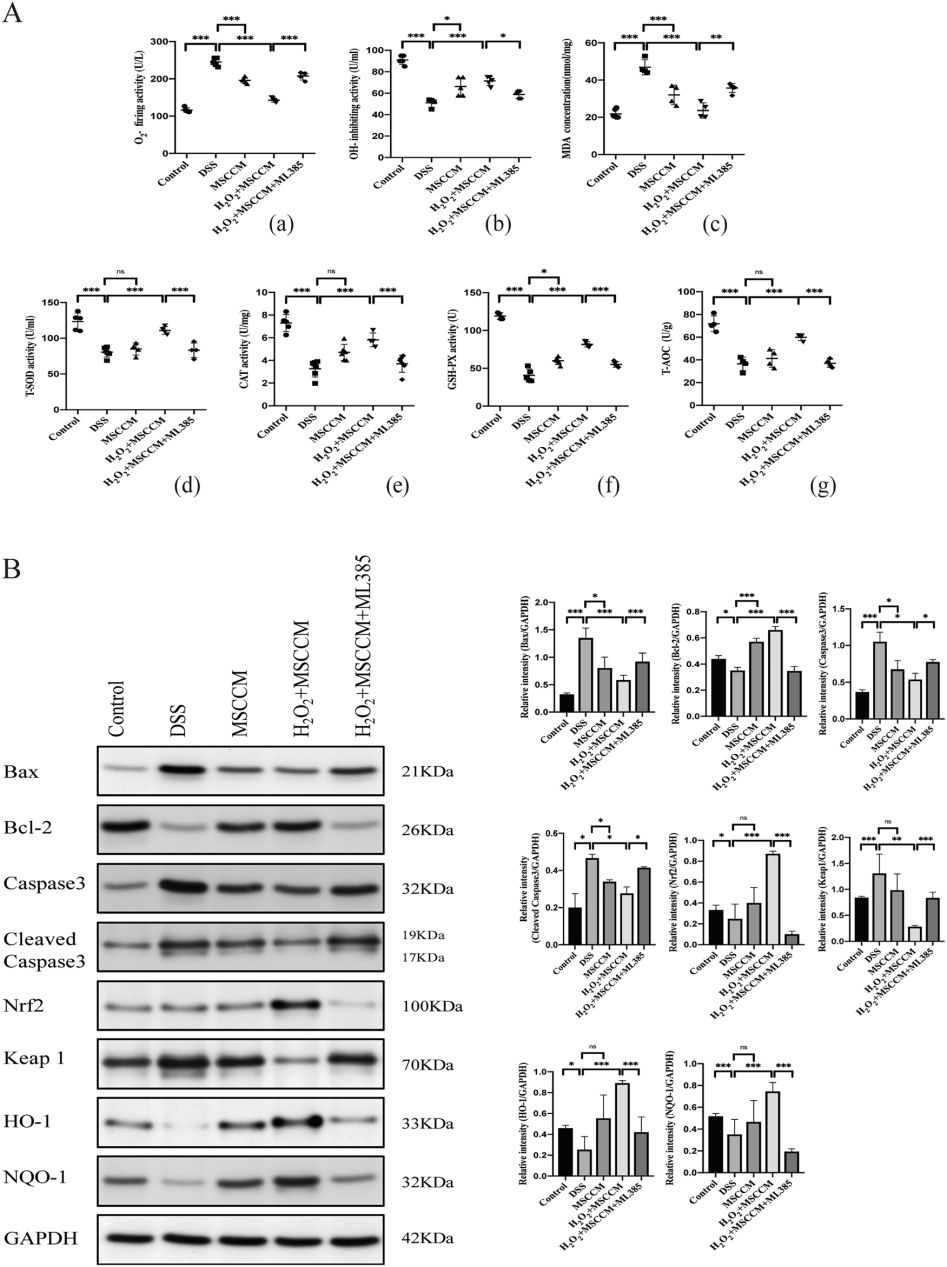
H_2_O_2_ pretreated MSC-CM exhibits anti-oxidant and anti-apoptosis activities in colon tissue. (**A**) The quantitative evaluation of oxidative stress in colon homogenate including O_2_^−^ (a), OH^−^ (b), and MDA (c). The indexes of the enzyme antioxidant system including SOD (d), CAT (e), GSH-Px (f), and T-AOC (g). (**B**) Immunoblotting assay determined the abundance of Bax, Bcl-2, Caspase 3, Cleaved Caspase3, Nrf2, Keap1, HO-1, and NQO-1 proteins. Semiquantitative analysis of proteins of interest by densitometry assay. The data are expressed as the mean ± SD. *P < 0.05, **P < 0.01, and ***P < 0.001; *ns* no significance.

We assessed some indicators of oxidative stress in mouse colonic epithelial cells, as shown in Fig. 7A. The activities of T-SOD, CAT, GSH-PX, and T-AOC in colon tissue in DSS-induced colitis mice were significantly decreased compared with those in mice in the control group; in contrast, the activities of O_2_^−^, OH, and MDA were increased. As expected, oxidative stress products such as O_2_^−^ and OH, and MDA activity were reduced after MSC-CM treatment. The T-AOC level, an indicator of the total antioxidant level composed of various anti-oxidative enzymes, was not significantly different compared with the DSS group. Administration of 25 μM H_2_O_2_-preconditioned MSC-CM reversed the DSS-induced changes, and when ML385 was added, the effective regulatory role in the oxidation reaction was inhibited.

Our above findings suggested a potent positive regulatory role for MSC-CM in anti-inflammatory and oxidative stress responses. To further explore the mechanism of oxidative damage in mouse colonic epithelial cells, we completed the apoptosis-related and Nrf2/Keap1/ARE signaling pathway-related proteins experiment (Fig. 7B). Compared with the control group, the DSS group was characterized by an increased abundance of the pro-apoptotic proteins Bax, Caspase 3 and Cleaved Caspase3 and decreased expression of the anti-apoptotic protein Bcl-2, and the differences were statistically significant. After MSC-CM enema treatment, the expression of apoptosis-related proteins was notably improved, implying that MSC-CM could restore colonic epithelial cell damage by inhibiting cell apoptosis. We also measured the levels of Nrf2/Keap1/ARE signaling pathway-related proteins to investigate the mechanism of anti-oxidative activity. Mice with DSS-induced colitis exhibited substantially decreased expression of Nrf2, HO-1, and NQO-1 and increased expression of Keap1 in colon tissues; however, the expression of these proteins was significantly improved only after 25 μM H_2_O_2_-preconditioned MSC-CM treatment. Notably, the presence of an Nrf2 inhibitor (ML385) blocked the therapeutic role of 25 μM H_2_O_2_-preconditioned MSC-CM in apoptotic responses and oxidative stress. Although without pretreatment, MSC-CM treatment effectively inhibited cell apoptosis, it had little effect on Nrf2/Keap1/ARE signaling pathway-related proteins.

## Discussion

The limitation of the use of MSCs is the reason why we shifted our attention to MSC-CM, and it is encouraging that MSC-CM can be used as an initially discarded product to achieve such satisfactory experimental results. According to the current research, the exact mechanism of MSCs is unclear, the paracrine effect of MSCs suggests that MSCs can produce bioactive molecules, exert anti-scarring, anti-inflammatory, and anti-apoptotic effects on target cells and surrounding inflammatory cells^22^. So we may have been focusing too much on MSCs and not enough on the role of MSC-CM.

Numerous studies have shown that pre-repeated short-term low-intensity damage stimulation can induce cells or the body to tolerate the subsequently sustained similar high-intensity damage stimulation, which is a ubiquitous adaptive protection phenomenon^23^. In this study, we used the oxidative stress model of MSCs induced by H_2_O_2_. In general, moderate levels of H_2_O_2_ may function as signals to promote cell proliferation and survival, playing a growth factor-like role in cells. Only a severe increase in H_2_O_2_ induces senescence and oxidative stress in MSCs^25^. In vitro, we confirmed that 25 μM H_2_O_2_ could maximize the proliferation and survival of stem cells without affecting their apoptosis. Therefore, we hypothesized that H_2_O_2_-pretreated CM could contain more secretions which include growth factors, cytokines, chemokines, and extracellular vesicles, to enhance anti-inflammatory and antioxidant stress ability and improve its therapeutic effect. Remarkably, we explored that the optimal concentration of H_2_O_2_ pretreatment MSCs was slightly different from the study of Guo et al.^13^. Their research results suggested that the optimal concentration of H_2_O_2_ pretreatment was 50 μM, which may be related to the type of MSCs and culture environment, but 25 μM and 50 μM were both acceptable and relatively low concentrations.

MSCs pretreated with 25 μM H_2_O_2_ can activate the Nrf2/Keap1/ARE pathway, which is the basis for subsequent animal experiments. When using DSS construction of experimental colitis model in mice, neutrophils and macrophages arrived in damaged regions of the intestinal mucosa and submucosa. Under the action of membrane DADH-coenzyme and oxidase, a large number of superoxide anion (O_2_^−^), hydroxy radical (OH), and lipid peroxides are produced through cellular respiration, which further induces the chemotaxis of neutrophils, lead to inflammatory infiltration of colon tissue and participate in the occurrence and development of IBD^27^. 25 μM H_2_O_2_-pretreated MSC-CM can inhibit the up-regulation of pro-inflammatory cytokines and oxidative stress in injured colonic epithelial cells by degrading Keap1 and activating Nrf2. The activation of the Nrf2/Keap1/ARE signaling pathway can affect the downstream antioxidant enzymes, including antioxidant enzymes catalase (CAT), total superoxide dismutase (T-SOD), glutathione peroxidase (GSH-PX), heme oxygenase (HO-1) and quinone oxidoreductase 1 (NQO-1) plays an essential role in anti-oxidative stress. As the presence of an Nrf2 inhibitor (ML385) eliminated the therapeutic role of MSC-CM in inflammatory responses and oxidative stress, this was confirmed laterally. In addition, we found changes in NF-κB P65 protein, which may also be related to the activation of the Nrf2/Keap1/ARE pathway. Activation of the Nrf2 pathway provides an endogenous defense system to balance the cytochemical microenvironment and influence some inflammatory signaling pathways, such as the NF-κB pathway^28^.

Anal injection (AI) administration is not common in clinical practice. However, for the model of ulcerative colitis constructed by DSS, the lesion site was confined to the colon. The enema method could make MSC-CM reach the damaged area more directly. According to the summary of previous studies^29^, intraperitoneal injection (IP) and intravenous injection (IV) therapy are no less effective than AI, suggesting that MSC-CM may achieve better treatment outcomes simply by changing the route of administration. In addition, our treatment frequency is relatively high in general controlled studies, which is attributed to the fact that MSC-CM is easy to obtain and less affected by culture environment. However, cell therapy requires a high number of cells, usually up to 1 × 10^6^–1 × 10^7^. To avoid the destruction of cell activity, the injection must be completed in a short time, which requires high experimental requirements. In short, the efficacy of pretreated MSC-CM has been enhanced, and MSC-CM also avoids the limitations of cell therapy, which might be a promising and practical approach for accelerating clinic translation of MSCs therapy. However, different types of mesenchymal cells, different ways of preconditioning cells, frequency of treatment, and route of administration all have significant differences in disease outcomes. Therefore, more systematic research on MSCs are needed.

## Conclusions

This study showed that the pretreatment of MSCs with 25 μM H_2_O_2_ was the best pretreatment protocol to obtain improved MSC-CM. 25 μM H_2_O_2_-pretreated MSC-CM enema treatment can protect the damaged colonic epithelial cells in experimental colitis, and the therapeutic mechanism is related to Nrf2/Keap1/ARE pathway. These data may contribute to the clinical application of MSC-CM.

## Supplementary Information

Supplementary Information.

## Data Availability

The data that support the findings of this study are available from the corresponding author upon request.
