# Supplementary material for: Conditioned medium of mesenchymal stem cells pretreated with H2O2 promotes intestinal mucosal repair in acute experimental colitis
Source: Sci Rep. 2022 Dec 1;12:20772. doi: 10.1038/s41598-022-24493-y (PMC9715703; doi:10.1038/s41598-022-24493-y)
Supplement: Supplementary file 1 — Supplementary Information. [file 41598_2022_24493_MOESM1_ESM.docx]

**The original, unprocessed versions of the western blotting experiment**

Fig.1. Immunoblotting assay determined the abundance of BAX proteins (Corresponds to Figure 1E, Figure 7B, and Figure 2B respectively).

Fig.2. Immunoblotting assay determined the abundance of Bcl-2 proteins (Corresponds to Figure 1E, Figure 7B, and Figure 2B respectively).

Fig.3. Immunoblotting assay determined the abundance of Caspase3 proteins (Corresponds to Figure 1E, Figure 7B, and Figure 2B respectively).

Fig.4. Immunoblotting assay determined the abundance of Cleaved Caspase3 proteins (Corresponds to Figure 1E, Figure 7B, and Figure 2B respectively).

Fig.5. Immunoblotting assay determined the abundance of Nrf2 proteins (Corresponds to Figure 7B and Figure 2A).

Fig.6. Immunoblotting assay determined the abundance of Keap1 proteins (Corresponds to Figure 7B and Figure 2A respectively).

Fig.7. Immunoblotting assay determined the abundance of HO-1 proteins (Corresponds to Figure 7B and Figure 2A).

Fig.8. Immunoblotting assay determined the abundance of NQO-1 proteins (Corresponds to Figure 7B and Figure 2A).

Fig.9. Immunoblotting assay determined the abundance of ZO-1 proteins (Corresponds to Figure 6C).

Fig.10. Immunoblotting assay determined the abundance of GAPDH proteins. (Corresponds to Figure 1E, Figure 7B, Figure 2A, Figure 6C, and Figure 2B).
